# Numerical Prediction of Local Instability in Double Corrugated Profiles

**DOI:** 10.3390/ma14206002

**Published:** 2021-10-12

**Authors:** Artur Piekarczuk, Przemysław Więch

**Affiliations:** Instytut Techniki Budowlanej Filtrowa 1, 00-611 Warsaw, Poland; p.wiech@itb.pl

**Keywords:** double-corrugated profile, buckling, validation, FEM

## Abstract

The technological process of forming the double-corrugated structures of the K-span system causes deep transverse embossing on the surface of the profiles. Such profile geometry makes it difficult to apply classical theories related to plastic failure mechanisms to identify the formation of local instabilities. This article presents an original method for the prediction of local instabilities of double-corrugated structures. The method was developed on the basis of a hierarchical validated FEM model. The geometrically and materially nonlinear analysis method was adopted to perform numerical calculations. The results of calculations enabled the determination of reference equilibrium paths for the eccentrically compressed shell element. Based on the analysis of nonlinear equilibrium paths, a method for predicting the beginning and the end of the appearance of local instabilities in the elastoplastic pre-buckling range was developed.

## 1. Introduction

**Figure 1:**
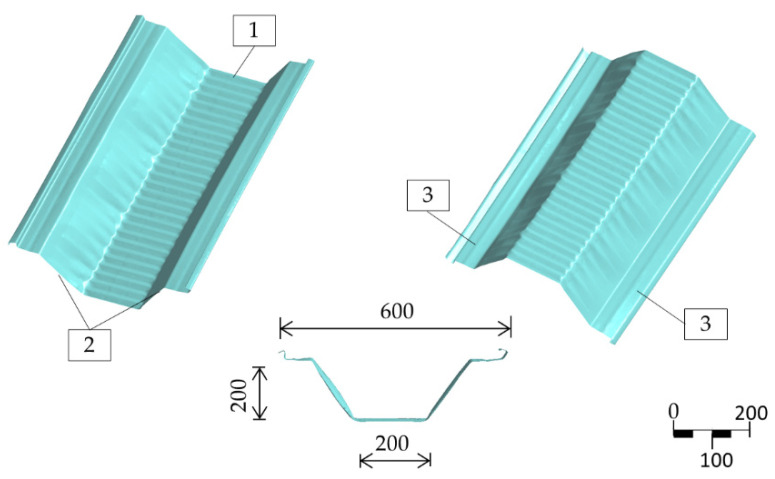
View of an ABM-240 profile fragment after rolling, dimensions in mm. 1—corrugated web, 2—corrugated flange, 3—flat lip.

**Figure 2:**
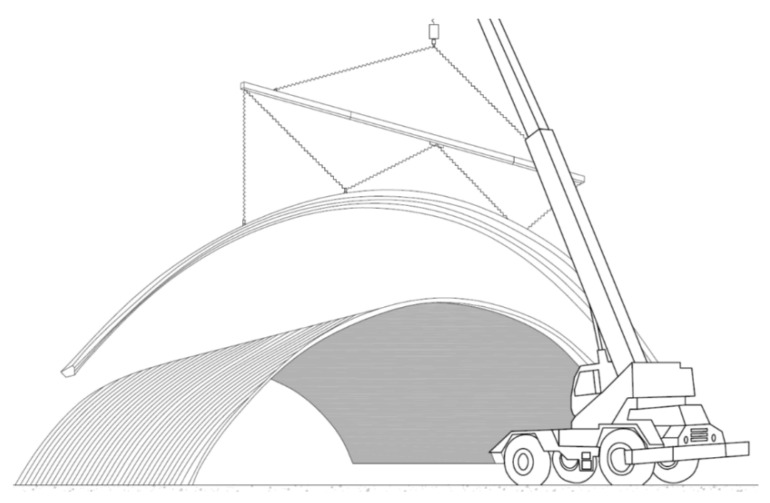
Assembly of an arch-shaped roof’s section.

Arch-shaped roofs made of bent metal sheets date back to the mid-twentieth century. Such roofs were mainly used as temporary military buildings [1,2]. The structure was light, quick and easy to assemble. The use of mobile rolling mills was a breakthrough. It was then possible to produce structural elements directly at the construction site. Since then, this construction type has become popular in civil engineering. The construction now known as K-span was initially used for agricultural and storage buildings. Over time, it has evolved to include residential and public utility construction. Several varieties of this system have been developed, with different shapes and dimensions of the trough’s cross-section as well as the assembly methods [3]. The MIC-240 ABM system is one of the most popular [4]. This system makes it possible to construct arched roofs with a span of up to 24 m. Long curved profiles are of cold-rolled steel sheets with a nominal thickness from 0.5 mm to 1.5 mm. A rectilinear section of a profile with a trough cross-section is made in the rolling process. In the next step the section’s surface is burnished and thus curved into a circular carve. As a result, curved double-corrugated profiles with characteristic deep embossing are created (Figure 1). Numerous curves and significant deformation of the side surfaces are characteristic of the so-called third-generation profiles [5]. Individual profiles are assembled into sections and mounted with a crane. After the assembly of sections, the roof forms a cylindrical segment (Figure 2).

The profile’s irregular shape makes it difficult to develop a mathematical description and impossible to qualify according to the known design standards [6,7,8]. There is currently no sufficiently good calculation method for this type of profiles. This situation often leads to inappropriate design, which may result in failures and catastrophes described, e.g., in an article by A. Biegus [9] and reported in the press [10,11,12].

A sketch of the assembly of K-span arch structures is presented in Figure 2. An example of actual assembly in field conditions is presented in the online access [13].

A number of dedicated scientific articles have been published with the attempt to examine and define methods for the design of K-span profiles. Publications on arched structures have been extensively discussed in the review by A. Piekarczuk et al. [14]. Some of the publications presented in this article are historical and are used to help understand the development of research techniques and calculation methods. They are of limited practical use today owing to the fact that manufacturing and assembly techniques have significantly changed. Although contemporary articles [15,16,17,18,19] cover wide research and a numerical system analysis program for K-span, these works, however, are focused on the ABM-120 variant, which is significantly different from ABM-240 in the cross-section’s shape and dimensions. These differences make it impossible to directly adopt these research results to the ABM-240 system’s assessment despite the identical manufacturing and assembly technology of arch structures.

The research and theoretical works on the ABM-240 system are covered in [20,21]. These publications present testing techniques for small samples and full-size elements and are focused on the causes and mechanisms of failure of the ABM-240 structure. In addition to the research and analytical works indicated above, the theory of load-carrying capacity and failure mechanisms related to thin-walled elements plays an important role in the discussion. In this case, publications by M. Kotełko et al. [22,23,24] prove to be useful, presenting the most important scientific achievements in the field. 

**Figure 3:**
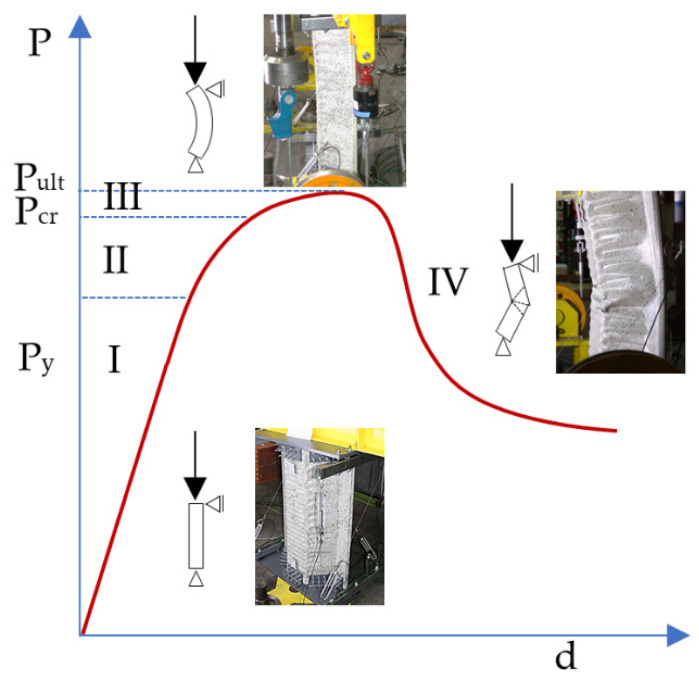
Shells’ behaviour. Buckling in the elastoplastic range. P_Y_—yield point, P_cr_—critical point, P_ult_—ultimate point, d—displacement. I—pre-buckling, II—pre-buckling elastoplastic range, III—post-buckling elastoplastic range, IV—failure.

The authors’ own research presented in [25] and based on the theoretical framework as described in M. Kotełko’s works covers the theoretical explanation of the causes of plastic buckling formation in double-corrugated profiles of the K-span ABM-240 system. This work explains what happens when a post-buckling elastoplastic range state is attained, that is in the third work phase of a double-corrugated thin-walled profile. The article also presents the method to determine the load-carrying capacity limit. Figure 3 presents the theoretical equilibrium path of the shell-type element. 

In the case of such complex geometry, however, the local instabilities formation mechanism remains unknown. It is necessary to study the equilibrium path for the pre-buckling elastic range (phase II, Figure 3) to explain the mechanism. The authors’ own research demonstrates that the first signs of local buckling appear in this phase of the section’s work; the structure has large displacements. The relationships between strain and displacements become non-linear while the strain–stress constitutive relationships remain in the linear range. 

The knowledge about the local instability formation mechanism is useful for predicting the entire structure’s stability and load-carrying capacity and particularly useful for spotting the nature and place of damage. Scientific studies on the subject [26,27,28,29] have made a significant contribution to the development of science and technology, but they are usually related to flat-walled profiles of regular geometry. Determination of the failure mechanism becomes more complicated with irregular geometry such as the double-corrugated one. Therefore, the search for buckling mechanisms carried out with classical methods [22,23,24,26,27,28,29,30] is difficult in the case of double-corrugated profiles. 

**Figure 4:**
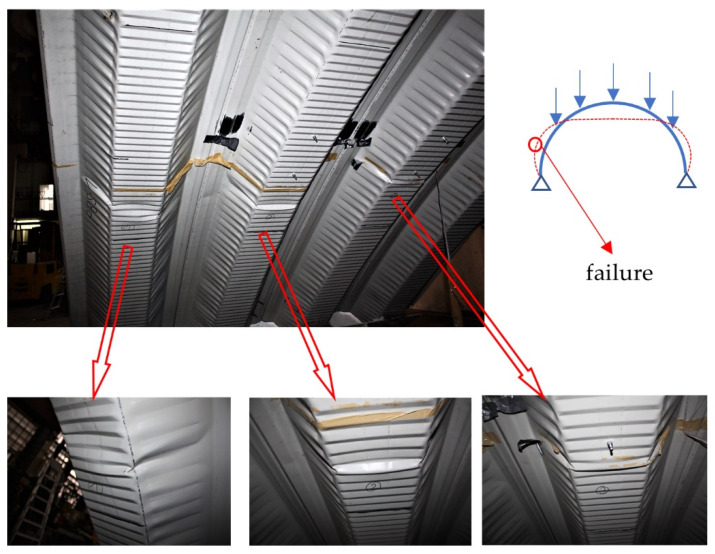
A characteristic mode of profile damage was recorded in the studies on full-size arched structures.

The authors’ previous research and analyses have demonstrated that damage most often occurs on the inner part of the trough section, in the cross-corrugated places (Figure 1). There is practically no damage to uncrimped surfaces; it may develop, however, as secondary damage (after the entire system loses its stability). Figure 4 shows characteristic damage to trough profiles that appeared during tests on full-sized arched structures [31]. A similar damage type and strong nonlinearity of the load–displacement relationship are indicated in [32,33,34,35]. This damage type results from the compressive stresses’ impact caused by eccentric loads. Such loads result in simultaneous occurrence of longitudinal forces and bending moments in the arched structure.

To investigate the mechanism which causes instability, the damage effects described above were reproduced on a smaller scale. The research methodology was presented in detail in [25,36].

In this article, the test results were used to assess the compatibility of the numerical model, which was then tested for the deformation course under the compressive force and bending moment in a typical damage pattern. The test results have two basic parameters only: force and a corresponding displacement. The calculation-based numerical data make it possible to determine forces, displacements and strains, stresses, etc., in the full field of observation (sample surface) with the evidential reading vector. Thus, a correct numerical model was assumed to be more useful than the results of the test based on a limited data pool. The numerical model was thoroughly validated and subjected to numerical tests. The numerical calculation results are used to search for the mechanism of local damage occurrence. 

The paper presents the detection method for buckling and local instability formation. The method is based on observation of equilibrium path nonlinearities in the phase II pre-buckling elastic range (Figure 3), that is, before the plastic range appearance. 

## 2. Methods

### 2.1. The Numerical Model’s Validation 

#### 2.1.1. Experimental Data 

**Figure 5:**
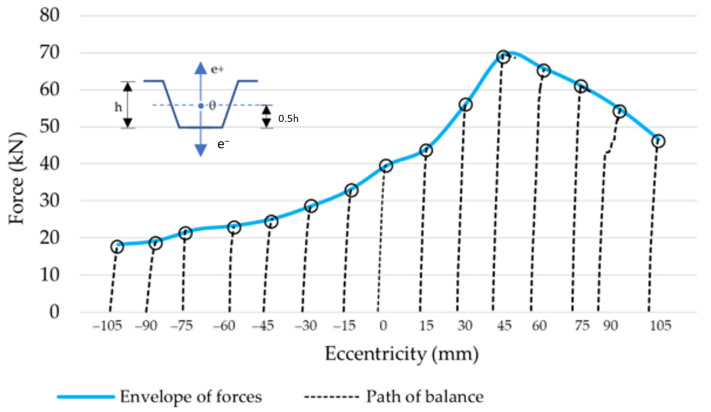
Results of eccentric compression tests.

In order to test local damage in phase II of the pre-buckling elastic range, a representative fragment of the ABM 240 system profile was selected that was the subject of the research presented in our own publication [36]. There were 15 tests in total consisting of fragments of the ABM 240 double-corrugated profiles subjected to eccentric compressive loads. The test specimens were 1.0 m long and cut out from a longer piece with an 18.0 m bending radius. The profile was made of a 1.0 mm thick steel sheet with the following strength parameters: yield strength fy = 337 MPa and ultimate strength fu = 387 MPa. The tests were performed on a special test stand described in [36]. Compared to [36], the analysis is much more comprehensive in this article as it includes the entire range of peak load values (maximum loads from each test series) and the selected equilibrium path of the model corresponding to typical damage. Figure 5 shows the equilibrium paths resulting from the 15 tests. Each equilibrium path is the test result of eccentric compression, with different eccentricity values in relation to the profile mid-height. The maximum loads recorded in each of the 15 tests constitute the control points. The line that connects these points constitutes the envelope of the load-carrying capacity, as shown in Figure 5. 

This envelope is used to verify the numerical model correctness in the whole range of eccentricity variability. The test results of the maximum forces at given eccentricities are presented in Table 1. 

**Figure 6:**
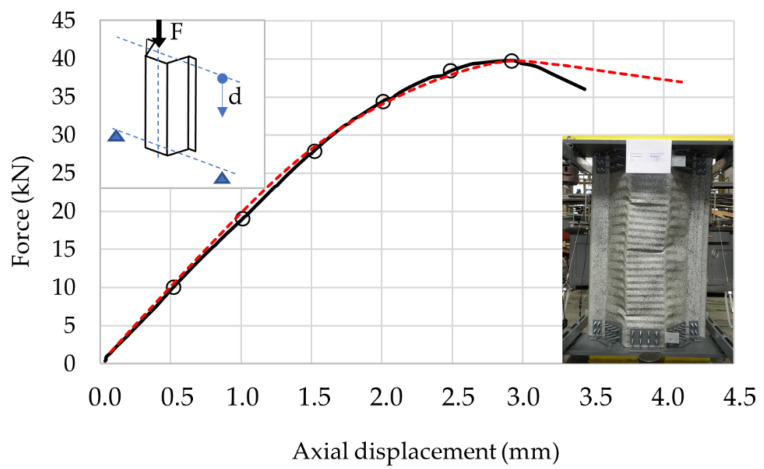
Model 0. An equilibrium force–displacement path at an eccentric compression.

The data obtained in compression with e = 0 mm were selected for the purpose of a detailed analysis of the load displacement equilibrium path of the sample during eccentric compression. With this eccentricity, a failure mode typical of this profile occurs, consisting in compression of the corrugated part of the profile web. This sample is referred to as Model 0 in the course of this paper. The equilibrium path resulting from the eccentric compression test at e = 0 mm with the number of checkpoints is shown in Figure 6. Figure 6 also presents a simplified load diagram and the direction of displacement measurement, as well as a photograph of typical damage.

The numerical values of the equilibrium path checkpoints are presented in Table 2. The last reference point represents the maximum force and the corresponding displacement. 

The data on the envelope (representing the entire range of eccentric compression) and the equilibrium path of a representative sample are the basis for the numerical model’s verification and validation. 

#### 2.1.2. Numerical Data

The geometry of the numerical model adopted for calculations was obtained by 3D scanning and it is an exact representation of the research element with real-life technological geometric imperfections. The model’s shape, together with the 3D surface geometry details, is shown in Figure 1. The geometric topology was imported into the ANSYS computing environment as point cloud data. Load and boundary conditions, as well as the material model, were created in the ANSYS numerical module. Most of the works were carried out in the same way as in [36]. Only minor defects of the 3D scan were corrected for the purpose of this article and some sectors were simplified by eliminating irregular shapes of the surface division. 

**Figure 7:**
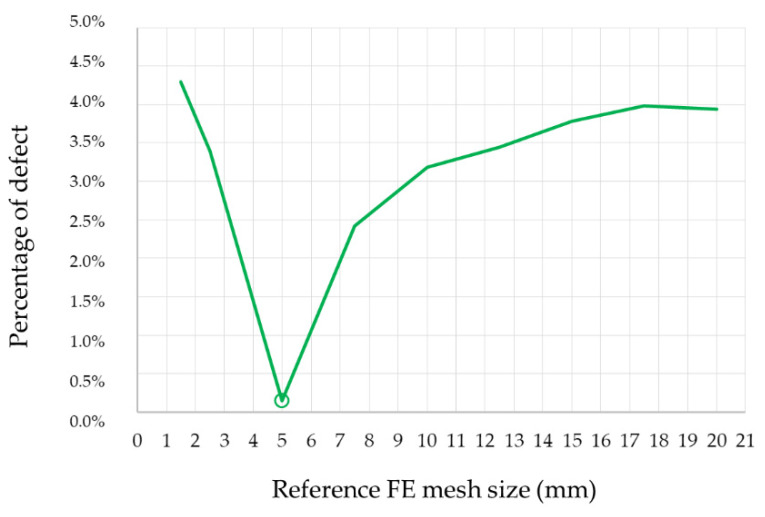
EC mesh optimisation error distribution.

The FE mesh was optimised before the calculations started. The standard ANSYS software methods can be used to estimate the FE mesh error: stress energy error (SERR), element stress deviation (SDSG), percentage error in energy norm (SEPC) and maximum and minimum stress bound (SMXB/SMNB) [37]. The energy method [38] is most commonly applied as an alternative to inertial methods. Comparative methods are also used, as in [39]. The above methods are used when there is no reference to laboratory test results. Since the research results presented in this article are known, a simple incremental method that relies on tests of error increments was adopted. The optimisation process consisted of a forced change in the finite element’s size, i.e., its reference dimension (D) [40], so as to obtain the peak value of compressive force as close as possible to the ultimate load obtained from the Model 0 sample tests. The change was forced in the EC grid parameters in the control module. The number of nodes obtained after the generation of the entire model mesh was an additional control parameter. The optimisation results summary is presented in Table 3. A graphical representation of the percentage error distribution of the force determined from Equation (1), depending on the ES reference dimension, is shown in Figure 7.

(1) $$\delta =\left|\frac{F_{test}-F_{FEM}}{F_{test}}\right|\times 100\;\left[\%\right]$$

where $F_{test}$—the ultimate load obtained from tests on the Model 0 sample = 39.768 kN (data from Section 2.1); $F_{FEM}$—the peak value of compressive force obtained from the FEM calculations with the assumed mesh parameters.

The optimisation test showed that the best convergence of results was obtained with the ES = 5.0 mm reference mesh size. The mesh size increasing up to 20 mm obviously increased the error. On the other hand, it is interesting to see the results obtained for a mesh smaller than 5.0 mm. With the reduced mesh size, the error turned out to increase. Finite element mesh irregularity was the most probable cause of such a situation. Curvature of deep corrugations necessitates adjustment of the finite elements’ topology to fit the complex shape of the modelled profile. A too dense or too sparse mesh results in irregularly shaped elements that have a negative impact on the FE solution.

As a result, the optimal mesh with a reference dimension of 5.0 mm was adopted for further numerical tests. The numerical simulation uncertainty (determined according to Equation (1)) due to the FE mesh optimisation is U_SN_ = 0.14%. 

#### 2.1.3. Hierarchical Assessment

**Figure 8:**
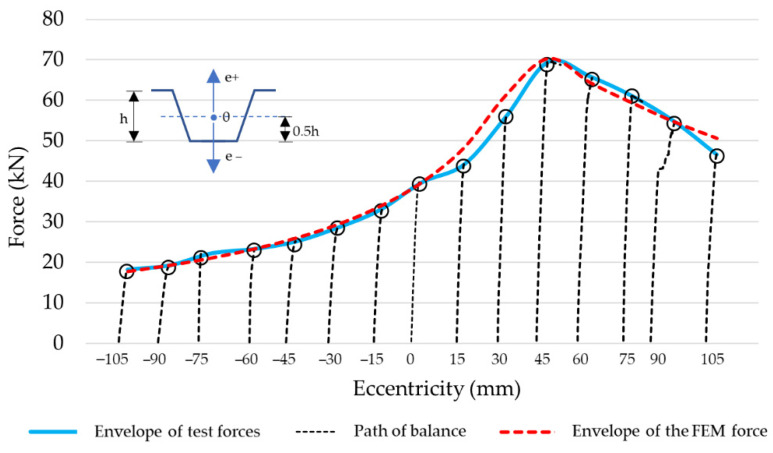
The graphical representation of the FEM versus the experimental test results at different eccentricities of compressive force.

**Figure 9:**
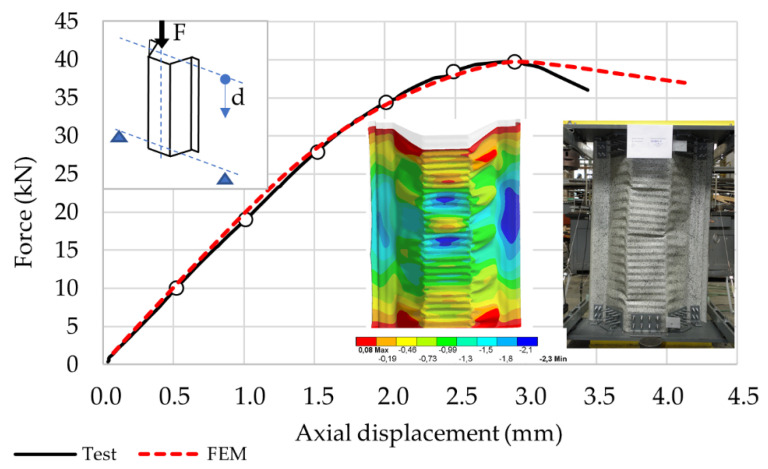
The graphical representation of the FEM versus the experimental test results for the Model 0 sample’s equilibrium path.

The optimised numerical model was subjected to further tests. The material model as in [36] was adopted for the calculations. The test’s purpose was to determine the error between the numerical model and the experimental test results. A series of calculations were performed to verify the maximum forces at varying eccentricity at the reference points shown in Figure 5 and Table 1, as well as the Model 0 sample equilibrium path at the reference points as in Figure 6 and Table 2. The test results are presented in Figure 8 and Figure 9. Detailed results related to the reference points are tabulated in Table 4 and Table 5, respectively. 

Experimental and numerical equilibrium patches diverged after the ultimate load level was reached, with a steeper decrease in the force value obtained from the Model 0 test. This phenomenon was caused by two main reasons. First, the differences existed in the numerical model and the experimental setup. In the FEM model, only the profile section was recreated, with the load applied directly to it. The experimental setup, however, included a number of additional components that stored a significant amount of elastic energy. While this factor is not important in the quasistatic test range, it plays a significant role during an abrupt failure of the tested specimen when the stored energy is released instantly. The second reason is that the Newton–Raphson method was used in the numerical calculations resulting in smooth convergence to the equilibrium path. 

Since this phenomenon occurred only after specimen failure, its importance was negligible from point of view of this article.

Validation is a hierarchical process of assessment of the numerical simulations’ reliability. In this case, this process metric was used [41] in the form of a function:

(2) $$\left|E\right|<U_{V}$$

where *E*—comparison error, *U_V_*—validation uncertainty. 

If condition (2) is met, then the validation is positive, which means that the numerical model is useful for further analyses.

The comparison error (*E*) is defined as the difference between the value obtained from the tests and that obtained from the numerical calculations. In this case, the previously determined values of the comparison errors were used in the envelope tests ($\delta_{e}$= 3.40%) and in Model 0 ($\delta_{M0}$= 2.32%):

(3) $$E=\sqrt{\delta_{e}^{2}+\delta_{M0}^{2}}$$

With the use of the numerical data calculated using Equation (3), the result was *E* = 4.11%.

The following equation determines validation uncertainty:

(4) $$U_{V}=\sqrt{U_{D}^{2}+U_{SPD}^{2}+U_{SN}^{2}}$$

where *U_SN_*—uncertainty in the numerical simulation, *U_SPD_*—uncertainty related to material input data, *U_D_*—uncertainty of experimental data. 

The uncertainty of the numerical simulation in the adopted model is presented in Section 2.1.2 and equals *U_SN_* = 0.14%. The uncertainty of the material input data and experimental data was assumed in accordance with [42] and equals *U_SPD_* = 3%. The uncertainty of the experimental data related to the force measurement for this study is presented in Chapter 7 of [3] and equals *U_D_* = 5%. Considering the numerical data calculated from using Equation (4), *U_V_* = 5.83 was obtained.

To conclude, $\left|E\right|=4.11\%<U_{V}=5.83\%$.

The numerical model adopted for the calculations was validated with a positive metric result, which means that the numerical model is useful for further parametric tests, and the calculation results will be affected by an error not greater than that in the results of the experimental tests.

### 2.2. Reference Numerical Model and Analysis Criteria 

The numerical model was divided into sections, for which reference points were selected in order to read displacements at various load stages.

**Figure 10:**
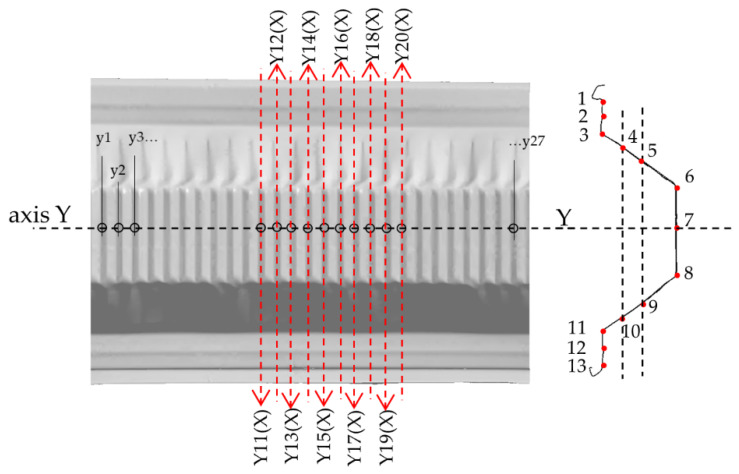
The reference points locations, Model 0.

One longitudinal section, symmetrically across the profile web (Y-axis), and seven cross-sections (Y(X)-axis) were chosen. There were 27 reference points in the Y-axis; each one was 37 mm away from the next point, which corresponds to the half-wave of the web’s corrugation. The cross-sections located at reference points from Y11 (X) to Y20 (X) in the area contained three full half-waves of local deformation. In each cross-section (Y(X)-axis), there were 13 reference points according to the diagram in Figure 10. Longitudinal sections and cross-sections are placed where a representative form of deformation is expected to occur. 

The test element was subjected to the reference loading stages with the use of an eccentric compressive force. The threshold values describing the consecutive stages are tabulated in Table 6.

**Figure 11:**
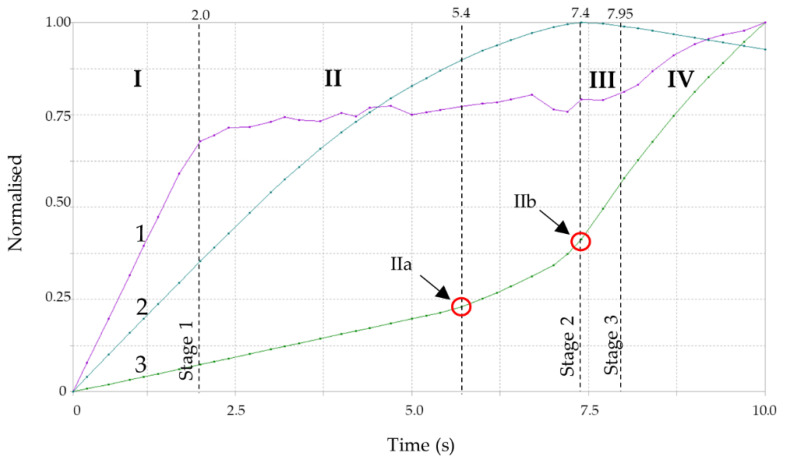
The course of control parameters in numerical calculations. 1—von Mises stress, 2—maximum displacement, 3—force. I, II, III, IV—the element’s work phases as described in Figure 3.

(a) Stage 1: geometry of the initial state of the phase I (pre-buckling elastic range). Stress below the yield strength.
(b) Stage 2: geometry of the phase II state (pre-buckling elasto-plastic range) at a load corresponding to the load-carrying capacity (critical point). This stage has two ranges, IIa (linear) and IIb, with the presence of strong nonlinearities. In range II, plastic buckling was formed, and there was an onset of a rapid deformation increase. The determination of the stresses initiating plastic buckling formation is illustrated in Figure 11.
(c) Stage 3: geometry of phase III state (post-buckling elastoplastic range) with the load in the plastic phase corresponding to the load-bearing capacity (ultimate point) when the ultimate strength is obtained. After exceeding phase III, the test element rapidly enters the fail state IV.

Reading of the displacement reference points took place at each load stage.

The calculations were performed with the use of the ANSYS software, assuming full nonlinearities, in accordance with the geometrically and materially nonlinear analyses with imperfections (GMNIA) methodology [8,43,44,45,46,47]. The test model was loaded the same way as the research element, i.e., a displacement until a clear ultimate point was reached.

The list of control parameters for the course of the calculation is shown in Figure 11. The control parameters pool included von Mises stresses (1), transverse displacements (2) and force, or rather a reaction to displacement (3). The horizontal axis scale (time) does not refer to the direct time measurement, but it shows the load increment course in successive nonlinear iterations of the computational solver. The vertical axis is the normalised reference value for each parameter. Normalisation is a dimensionless quantity, determined as the ratio of the parameter’s maximum value from the entire analysis (final value) related to the parameter value in the iteration course over time. The figure is divided into four sections separated by the load stages defined in 2.2. The separated sections correspond to the individual work phases of a thin-walled structure (Figure 3).

## 3. Results

### Test Results

**Figure 12:**
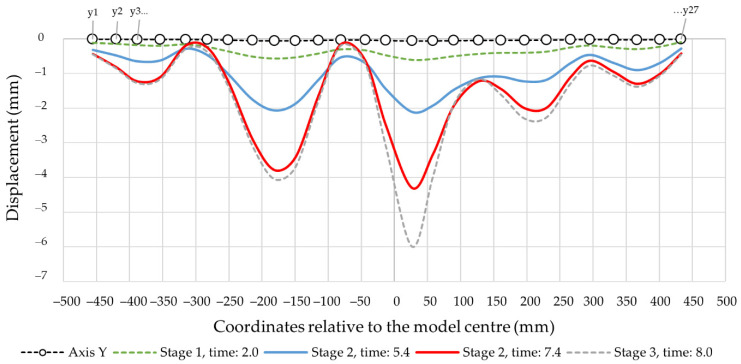
The deformation envelopes of the control points in the Y-axis. Global system.

Figure 12 presents the results of the calculations for the displacement envelope of control points in the longitudinal section (Y-axis). The envelopes relate to the reference ranges defined earlier:

- stage I, time: 2.0 (linear range);
- stage IIa, time: 5.4 (pre-buckling linear elastoplastic range);
- stage IIb, time: 7.4 (pre-buckling nonlinear elastoplastic range);
- stage III, time: 7.95 (post-buckling elastoplastic range).

**Figure 13:**
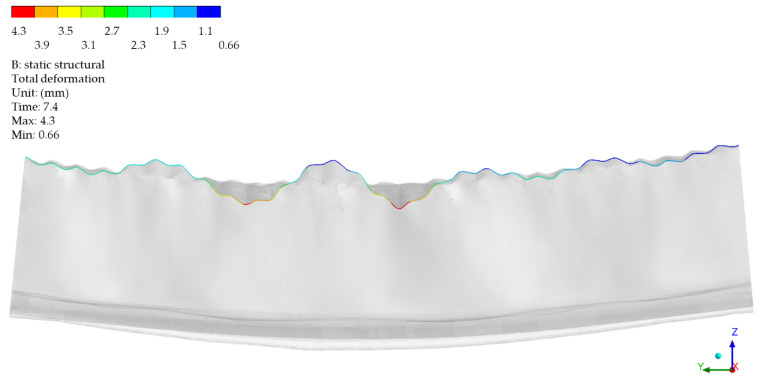
The deformation lines of the numerical model in the longitudinal section at stage 2, time: 7.4.

**Figure 14:**
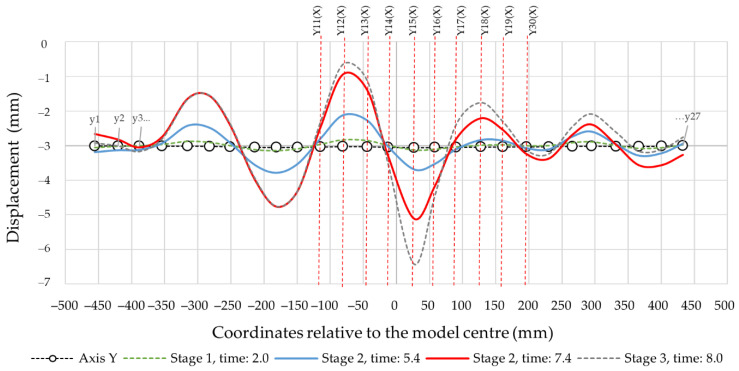
The deformation envelopes of the control points in the Y-axis. Local system.

The envelopes in Figure 12 are related to the global reference frame, which means that apart from the local deformations, its components contain the global displacement component, i.e., the element axis’ deflection, which increases along with the load. Figure 13 shows the deformation lines of the numerical model in the longitudinal section at stage 2, time: 7.4. The deformation shape corresponded to the data in Figure 12. Figure 14 demonstrates the same data, however, with local deformations only. Figure 14 demonstrates the cross-sections corresponding with the designates in Figure 10. Loss of stability occurred in section Y15 (X) which was shifted by 55.5 mm in relation to the longitudinal Y-axis’ centre. The half-waves length in the measurement area (between the transverse axes) was as follows: Y14(X) − Y11(X) = 101 mm, Y17(X) − Y17(X) = 102 mm and Y17(X) − Y20(X) = 106 mm.

**Figure 15:**
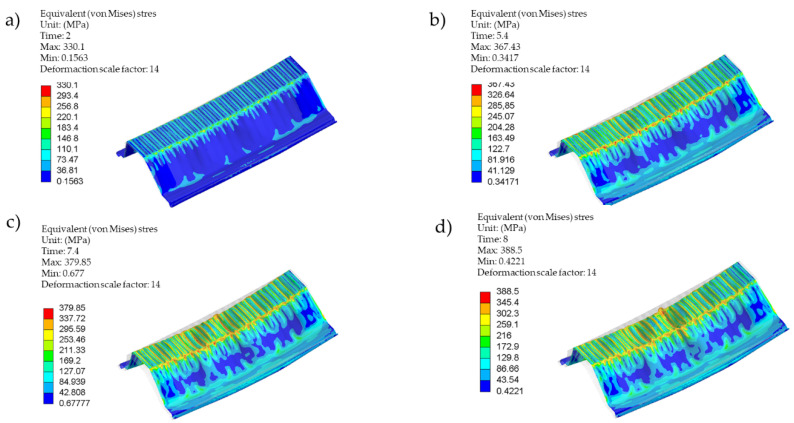
The stress maps and the test model deformation at the reference load stages, (**a**) stage 1: time 2.0, (**b**) stage 2: time 5.4, (**c**) stage 2: time 7.4, (**d**) stage 3: time 8.0.

Figure 15 shows the stress maps along with the reference element’s deformation in Model 0 at individual loading stages, i.e., the phases I, IIa, IIb, III. The stress maps of phases IIb and III are almost identical (Figure 15c,d). The difference is that the phase III deformation was much more pronounced. 

**Figure 16:**
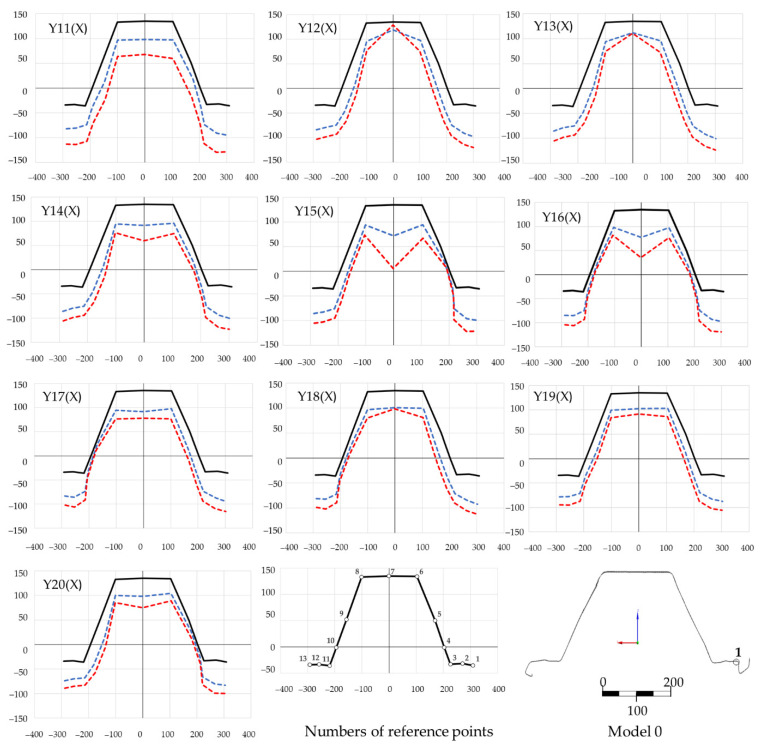
The cross-section deformations, two stages of loading: phase IIa (time: 5.4) and IIb (**blue line**), time: 7.4 (**red line**).

**Figure 17:**
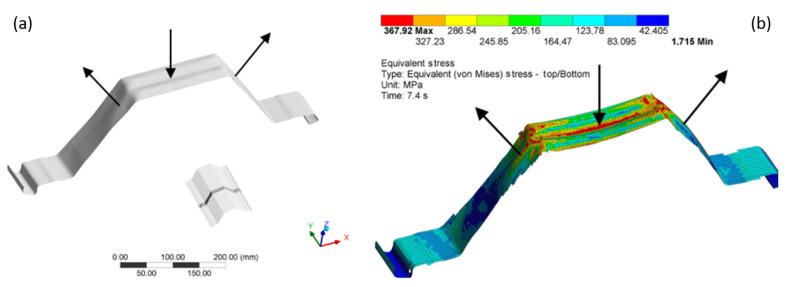
Profile surface’s geometry: (**a**) directions of surface bends, (**b**) von Mises stresses (phase IIb).

Figure 16 demonstrates the cross-sections’ deformation (Figure 15) in two loading stages: phases IIa and IIb. Plastic buckling form and develop in this load range. Plastic buckling formed and developed in the cross-section Y15(X) (Figure 16). Extremes of the local half-wave’s buckling are demonstrated in Figure 14. Figure 17 demonstrates a fragment of a deep corrugated profile section deformation. The wall surface: the flange is alternately convex and concave, similar to the web surface. Both wavy surfaces connect at the corners in such a way that the convex flange surface becomes the concave web. 

Figure 15c,d demonstrates the stress concentrations in the profile’s corners. Figure 17b demonstrates a detailed stress map of the profile section, taking into account the directions of surface bending. A change in the direction of surface bending at the profile’s corners causes stress concentration accumulation. 

## 4. Discussion

A large part of the article was devoted to the hierarchical assessment of the numerical model’s reliability. The assessment is a troublesome but extremely important endeavour. According to this publication authors’ opinion, this data preparation stage cannot be simplified or even omitted. The numerical model’s validation is vital because the results of FEM calculations are subject to detailed analyses presented later in the article and used to draw the key conclusions. 

Reliability is understood as the degree of confidence in the obtained results; the reliability assessment for calculations belongs to the two categories. The first, referred to as verification, is about the correctness of the mathematical apparatus used to describe a physical phenomenon, e.g., the complexity of differential equations or matrix records and their possible quality in a mathematical sense. In the case of FEM numerical methods, such verification is performed by testing the correctness of the mathematical description, numerical codes and the computing systems’ efficiency in relation to the numerical patterns generated in the so-called benchmarks, such as in the procedures carried out by NAFEMS [48]. The other category, referred to as validation, is about verifying the calculation results’ compliance with the test results of a physical phenomenon study. Taking into account the complexity of physical phenomena and the imperfect numerical methods used to describe the phenomena, adopting general assumptions and regularities proposed in [49,50,51,52] makes it easy to navigate in this domain. Validation and verification are often confused and improperly applied. This article uses a typical validation with process metric indicators proposed in [41]. Positive assessment of the validation process made it possible to use the numerical model for further conceptual work. 

The first observation that arises after the review of the literature data [22] and the results of the calculations analyses is that there is a concordance of the theoretical description (Figure 3) with the results of the analysis of the equilibrium paths (Figure 11). However, the equilibrium path specification is necessary to describe the behaviour of the test element. 

Because of the complicated profile shape (deep corrugations on the surface), an indirect method for detection of buckling and local instabilities formation was employed. The method is based on the observation of equilibrium path nonlinearities in the phase II pre-buckling elastic range instead of the classic approach [22,23,24,26,27,28,29,30] that relies on the determination of the plastic hinges’ geometry. Phase I is a pre-buckling elastic range and ends when the yield strength f_y_ = 337 MPa is achieved, transiting to the phase II pre-buckling elastoplastic range. The displacements in phase I were linear, and the stresses remained elastic (Figure 15a). The lateral displacements of the profile’s web were limited (Figure 14, stage 1). Figure 11 illustrates the equilibrium paths detailing the control parameters, i.e., stress (1), force (2) and displacement (3). The stresses (1) from phase I-t transformed into the plastic ones, but the deformations and force increase (2) and (3) remained linear initially. Then, with increasing load, they became nonlinear. Phase II had complex implications and transitions between nonlinear ranges. Phase II began at the moment of transition from elastic range I to plastic range II (after exceeding the f_y_ = 337 MPa yield strength). The rapid deformation increase starts the plasticisation development in phase IIa, corresponding to the von Mises stress σ_time:5.4_ = 367.43 MPa and ends when the extreme force in phase IIb is reached under the stress σ_time:7.4_ = 379.85 MPa. Rapid phase changes were also noticeable in plastic strain (Table 6) because plastic strains in phase IIb increased more than three times compared to phase IIa, while elastic strain remained at a similar level. It is worth noting that the entire phase II (IIa and IIb) took place within the stress range from 367.43 MPa to 379.85 MPa, i.e., within the plastic range (Figure 15b,c). The maximum force in phase IIb was achieved in the plastic range and amounted to 39.764 kN. The phase IIa and IIb deformations’ course and development in the referenced longitudinal section are illustrated in Figure 14 for the cross-sections in Figure 16. Phase IIa initiated plastic buckling, and its development continued to phase IIb, which was the critical point; once this point was crossed, the physical relations describing the stresses and strains state became nonlinear. A very small range of stress increase was observed in phase III, i.e., from σ_time:7.4_ = 379.85 MPa to σ_time:7.95_ = 387 MPa. Not the force, but the corresponding stress limit, which corresponds to ultimate strength f_at_ = 387 MPa, was the characteristic extreme of phase III. The force in phase III maintained the value of the phase IIb force, while the plastic strain was more than two times greater than the value obtained in phase IIb. This means that the plastic buckling in phase III was already well-developed, and rapid propagation was observed. After crossing the ultimate strength f_at_ = 387 MPa, the transition to a phase IV failure started. This phase’s characteristics were a sharp increase in displacement and a significant decrease in force. A secondary redistribution of plastic buckling in the next most strenuous regions of the profile was also possible. 

The analyses indicate that in this case, the local plastic buckling can be identified by following the equilibrium path of the reference parameters: stress and displacement as a force increment function. The plastic buckling development occurred in phase II. In order to properly identify the onset and end of the plastic buckling development, phase II should be divided into two ranges: the onset of the plastic buckling development occurred in the phase IIa pre-buckling linear elastoplastic range and expanded until reaching the phase IIb pre-buckling nonlinear elastoplastic range. In the phase III range, plastic buckling developed further until the critical point was reached. Once this point was crossed, there was the transition to the state IV failure and final profile destruction. 

It is also worth noting that the profile geometry influences the manner of its destruction. As mentioned earlier, the local stability loss did not occur at the geometric centre of the profile’s longitudinal axis, probably caused by the irregular shape of the profile surface embossing. Surface rolling caused deep embossing that alternately occurred on the web and flange surfaces; both surfaces connected at the corners in such a way that the flange’s convex surface becomes the concave web. Irregular stress concentrations were formed on edges, as shown in Figure 15c,d. Such alternate and irregular geometry continued along the entire profile length, depending on its bend radius and the thickness of the sheet. In some regions, the convex surface turned into a concave one at the same height (Figure 17a); there was stress concentration in such places, as shown in Figure 17b. The analyses show that this factor contributes to secondary propagation of plastic buckling. This means that buckling was formed in the central web area, as in the diagrams in Figure 16. Then, the development continued, especially in the phase IIb and phase III ranges, a rapid redistribution in the corners started, as in Figure 15c. As a rule, this phenomenon is a typical failure pattern, described in Section 1 (Figure 4).

## 5. Conclusions

The mechanisms of local stability loss in third-generation double-corrugated profiles are difficult to establish on the basis of traditional theories of plastic failure mechanisms because of the profiles’ complex geometry—curved along their axis, with deep transverse ribs and complex geometry and arrangement. 

The laboratory tests on profile samples provided insufficient data for a comprehensive analysis of the formation course of local instabilities. Therefore, a numerical profile model was prepared for the analysis, which accurately reflects the model’s geometry, followed by the hierarchical validation of the model, which was used for the comprehensive analysis. 

The article presents the method to detect instability formation spots. The method consists of the equilibrium path analyses and the detection of nonlinearity limits in the pre-buckling elastic range of phase II thin-walled structures. The detected phases are marked with the IIa and IIb symbols; they indicate the onset and the end of formation of the plastic buckling mechanism, respectively. The local stability loss starts in the profile web and ends at the corners where the concave and convex surfaces come together. 

The presented local instability analysis case represents the majority of the damage to typical arched structures (circular arch supported at two extremes), rendered with double-corrugated K-span ABM 240 sheets. In addition to typical structures, engineering includes structures with various load models and support methods, including K-span systems. In such cases, the local stability loss model may differ. Further research and conceptual work will continue, including atypical damage cases.

## Figures and Tables

**Table 1:** List of the maximums of forces and the corresponding eccentricities.

Force, *F_test_* (kN)	Eccentricity, e (mm)
18.201	−105
19.219	−90
22.201	−75
23.260	−60
25.119	−45
28.570	−30
32.936	−15
39.768	0
44.190	15
56.101	30
69.561	45
65.898	60
61.050	75
54.760	90
46.644	105

**Table 2:** Model 0 sample: the equilibrium path’s control points.

Force, *F_test_*_, *M*0_ (kN)	Displacement, d (mm)
0	0.00
9.645	0.50
18.860	1.00
27.553	1.50
34.345	2.00
38.481	2.50
39.768	2.92

**Table 3:** Parameters of ES mesh optimisation.

D (mm)	*F_FEM_* (kN)	Nodes	*F_FEM_*/*F_test_*	*δ* (%)
1.5	38.061	393.856	0.957	4.30
2.5	38.420	143.352	0.966	3.39
5.0	39.714	40.034	0.999	0.14
7.5	40.733	22.852	1.024	2.42
10	41.035	18.640	1.032	3.18
12.5	41.141	17.442	1.035	3.45
15.0	41.273	17.002	1.038	3.78
17.5	41.352	16.827	1.040	3.98
20.0	41.337	16.786	1.039	3.94

**Table 4:** The tabulation of the calculation versus the experimental test results at different eccentricities.

Eccentricity, e (mm)	*F_test_* (kN)	*F_FEM_* (kN)	*δ_e_* (%)
−105	18.201	17.698	2.77%
−90	19.219	19.228	0.05%
−75	22.201	21.043	5.22%
−60	23.260	23.241	0.08%
−45	25.119	25.944	3.29%
−30	28.570	29.355	2.75%
−15	32.936	33.797	2.62%
0	39.768	39.764	0.01%
15	44.190	48.342	9.40%
30	56.100	61.388	9.43%
45	69.561	70.247	0.99%
60	65.898	64.236	2.52%
75	61.050	59.217	3.00%
90	54.760	54.563	0.36%
105	46.644	50.566	8.41%

The mean comparison error was *δ_e_* = 3.40%.

**Table 5:** The tabulation of the calculation versus the experimental test results for the Model 0 sample’s equilibrium path.

Displacement, d (mm)	*F_test_* (kN)	*F_FEM_* (kN)	*δ_M_*_0_ (%)
0	0	0	0
0.50	9.645	9.852	2.15%
1.00	18.860	20.070	6.42%
1.50	27.553	28.385	3.02%
2.00	34.345	34.011	0.97%
2.50	38.481	37.965	1.34%
2.92	39.768	39.764	0.01%

The mean comparison error was *δ_M_*_0_ = 2.32%.

**Table 6:** Threshold values: shells’ behaviour.

Stage	Time	*F_FEM_* (kN)	ε_el_ × 10^−3^	ε_pl_ × 10^−3^	σ (MPa)	σ/f_y_
I	2.0	15.023	1.644	0.00	337.00	1.000
IIa	5.4	34.778	1.878	7.804	367.43	1.090
IIb	7.4	39.764	1.967	23.864	379.85	1.127
III	7.95	39.763	2.106	48.572	387.01	1.153

ε_el_—elastic strain range, ε_pl_—plastic strain range.

## Data Availability

Not applicable.
